# The Development of Our Organ of Other Kinds—The Gut Microbiota

**DOI:** 10.3389/fmicb.2016.02107

**Published:** 2016-12-23

**Authors:** Shirong Liu

**Affiliations:** Department of Neurology, Ann Romney Center for Neurologic Diseases, Brigham and Women's Hospital, Harvard Medical SchoolBoston, MA, USA

**Keywords:** host-microbe interactions, microbiota, microRNA (miRNA), interkingdom interactions, antimicrobial peptides, antibiotic resistance

Gut microbiota is a collection of microbial organisms that naturally exist within the gastrointestinal (GI) tract. It is clear that the gut microbiota is involved in host physiological development, defense against pathogens and diseases. Thus, gut microbiota is regarded as a hidden organ composed of other kinds (Bocci, 1992). A feature of the gut microbiota is that the compositional microbes are host species-specific. For a given host, a defined core microbes exist which distinguish one host from another (Kostic et al., 2013). For example, Hawaiian bobtail squid selectively acquires *Vibrio. fischeri (V. fischeri)* from environment and maintains them in its light organ. This symbiont is so selective that other *V. fischeri* strains, such as a fish-associated *V. fischeri* strain are not able to colonize the squid (Mandel et al., 2009; Kostic et al., 2013). Zebrafish gut is predominated by members of the γ*-Proteobacteria* and *Fusobacteria* classes (Kostic et al., 2013). Wild mouse gut is primarily composed of *Bacteroidetes, Firmicutes, Tenericutes*, and *Proteobacteria* (Hoy et al., 2015; Weldon et al., 2015; Xiao et al., 2015). Interestingly, transfer of the conventional zebrafish microbe to germ-free (GF) mice did not result in a zebrafish-like gut microbiota in the recipient mice; vice versa, conventional mouse gut microbe-transplanted GF zebrafish did not resemble the gut microbes of mice. Rather, the gut microbiota of both recipients after transplantation resembled the microbiota of their conventional species. Thus, the host gut predefined its microbiota (Rawls et al., 2006). While host-microbe interaction is a two-way crosstalk (Celluzzi and Masotti, 2016), in this opinion article, I wish to discuss intrinsic factors that contribute to the development of gut microbiota. Namely, where the microbes come from and how the host selects them.

## The source of microbiota

Studies of infants prior to delivery and shortly after birth showed that meconium microbiota shares features with the microbiota in the placenta, amniotic fluid, and colostrum, suggesting a route by which the fetus obtain microbiota (Collado et al., 2016). At the age of 3–4 days, the infant gut microbiota composition resembles that detected in colostrum. Thus, a stepwise microbial gut colonization process may be initiated prenatally by a distinct microbiota in the placenta and amniotic fluid (Collado et al., 2016).

The microbiota link between the mother and offspring is continued at and after birth by microbes present in birth canal, maternal feces, and skin during delivery; as well as postnatal breast milk, skin contact, and environmental exposure. Vaginally delivered infants harbor microbes resembling their mother's vaginal microbiota, dominated by *Lactobacillus, Prevotella, or Sneathia* spp., while cesarean-section infants have microbial feature similar to that of their mother's skin and hospital environment, dominated by *Staphylococcus, Corynebacterium, and Propionibacterium* spp (Dominguez-Bello et al., 2010; Kostic et al., 2013). Feeding mode is another strong factor that affects infant gut microbiota development. A major bacterial source for the infant gut is the maternal milk. Two main bacteria *streptococci* and *staphylococci* in the breast milk (Heikkilä and Saris, 2003; Mandel et al., 2009; Kostic et al., 2013) are among the earliest colonizers of the infant gut (Palmer et al., 2007; Kostic et al., 2013; Avershina et al., 2014). After birth, as time increases and the host keeps exposing to different microbes, the intestinal microbiota transits rapidly from less diverse with a relative dominance of the phyla *Proteobacteria* and *Actinobacteria* to more diverse with dominance of *Firmicutes* and *Bacteroidetes* (Bäckhed, 2011; Hoy et al., 2015; Weldon et al., 2015; Xiao et al., 2015). By 3 years of age, the diversity and composition of gut microbiota resemble those of adult human and become relatively stable (Rawls et al., 2006; Yatsunenko et al., 2012; Faith et al., 2013).

The availability of microbes determines which microbes have the chance to colonize the gut. Environmental impacts (including antibiotic use, geographical location, food, and life style, etc.) on the microbiota seem to be cumulative across generations (Rodríguez et al., 2015; Collado et al., 2016). The diversity of microbiota in the population of the US and earlier modernized countries is lower than that in the late modernized countries (Blaser, 2016; Collado et al., 2016). Antibiotic exposures during early life not only alter bacterial diversity but also delay microbiota maturation (Bokulich et al., 2016).

## Host genetic background endorses the gut microbiota

A successful arrival of a microbe in the GI tract does not guarantee colonization. For example, a systematic review of evidences suggests that the use of probiotics does not change the composition of fecal microbiota (Kristensen et al., 2016), suggesting that the host has an intrinsic selection mechanism.

Indeed, twins microbiota studies showed that monozygotic twin pairs had more similar microbiota as compare to dizygotic twin pairs (Goodrich et al., 2014). Investigators found that heritable taxa were the most stable taxa in TwinsUK dataset. Furthermore, the relative abundances of the heritable genus *Bifidobacterium* were associated with genetic variants in the genomic locus containing the gene *LCT* (Goodrich et al., 2016). A large cohort study evaluating the association between healthy host genetic variation and the composition of microbiota found that almost one-third of fecal bacterial taxa were heritable. Specific SNPs were associated with the relative abundance of specific taxa (Turpin et al., 2016). Thus collectively suggests that the host genetic background endorses the selection of gut microbial component.

Indeed, as discussed above, reciprocal gut microbiota transplantation in which the gut microbiota of adult GF mice colonized with an unfractionated gut microbiota harvested from conventional zebrafish was compared with GF zebrafish larvae colonized with a gut microbiota from conventional mice. Gut microbiota of zebrafish and mice share six bacterial divisions, although the specific bacteria within these divisions differ. The transplanted community resembled its community of origin in terms of the lineages present, but the relative abundance of the lineages resembled the normal gut microbial community composition of the recipient host. This study clearly demonstrated that whether a bacterial species could be potentially found in a host is determined by the chance of the host acquires that bacteria; and the bacterial community structure, in terms of species and abundance of a particular species, is shaped by the host habitat (Rawls et al., 2006).

## Pathways in which the host shapes microbes

When microbes pass through the GI tract, an environment they have to encounter is pH. A study of the gut microbiota in a cohort of 1827 healthy twins identified a significantly lower gut commensals and lower microbial diversity in proton pump inhibitor users, suggesting that pH in the gut impacts on the gut microbiota (Jackson et al., 2016).

Comparison of MyD88-, TLR2-, TLR4-, TLR5-, and TLR9-deficient mice and their respective wild-type (WT) littermates demonstrated that the impact of TLR deficiency on the gut microbial composition is minimal under homeostatic conditions and after recovery from antibiotic treatment (Ubeda et al., 2012), suggesting the innate recognition of microbes is not essential for commensals development. However, autonomous innate antimicrobial components do affect the microbial community. Antimicrobial peptides (AMPs) are ancient defense mechanism found in virtually every multi-cellular organism. They help the host to exclude a broad-spectrum of microbes. These peptides are produced by specialized gut epithelial cells and circulating inflammatory cells. Most prominent AMPs in the intestinal tract are represented by defensins, cathelicidins (e.g., LL-37), C-type lectins (such as the regenerating islet-derived protein (REG) family) (Cash et al., 2006), ribonucleases (RNases) and S100 proteins (e.g., calprotectin). Different AMP has different antimicrobial activity. For example, specialized Paneth cells in the small intestine store and secrete various antimicrobial effectors (e.g., lysozyme, phospholipase A2 group IIA or REGIII); but their most abundant products are the α-defensins human defensin (HD) 5 and HD6. Both α-defensins and β-defensins are bactericidal, with activity against Gram-negative and Gram-positive bacteria (Bevins and Salzman, 2011; Ostaff et al., 2013). It was reported that human gut microbes from all dominant phyla are resistant to high levels of Cationic AMPs polymyxin B. The investigators thus hypothesize that AMP is a mechanism that distinguish commensals from pathogens for colonization (Cullen et al., 2015). Host defense-related ribonuclease, such as murine RNase angiogenin 4 has the bactericidal activity both against Gram-negative and against Gram-positive bacteria (Bevins and Salzman, 2011).

The adaptive immunity affects gut microbiota as an altered microbiota was observed in immunodeficient Rag1^−/−^ mice. The bacterium *Akkermansia muciniphila* was highly enriched in Rag1^−/−^ mice compared with the wild type. This enrichment was suppressed when Rag1^−/−^ mice received bone marrows from wild-type mice (Zhang et al., 2014). As part of adaptive immunity, polymeric IgA from intestinal plasma can be secreted into the lamina propria and then transported into the gut in the form of secretory IgA (SIgA). SIgA may shape the gut microbiota through two mechanisms: in one 'immune exclusion' mechanism it interacts with antigens including microbes and limits the access of intestinal antigens to the blood circulation and thus controls the intestinal microbiota. The second mechanism is synergies between the SIgA and innate responses of the intestinal epithelium. SIgA can limit innate responses against commensal bacteria. Lack of SIgA results in enhanced stimulation of innate responses in gut epithelial cells (Peterson et al., 2007; Pabst, 2012).

While the above discussed mechanism act in a negative (punitive) selection way, recent found mechanisms arise as positive selection on the microbiota.

Foster group proposed a positive control model, in which a host acts in a way that promotes beneficial microbes, rather than inhibits harmful ones. In this individual-based “selectivity amplifier” model, epithelial secretions permeate upwards the whole microbial community, while lumen compounds preferentially affect cells that are soon to slough off (Schluter and Foster, 2012). They proposed that modest amounts of moderately selective epithelial secretions cause a complete shift in the strains growing at the epithelial surface. Possible molecules for such selection include epithelial-derived nutrients, such as fucose (Schluter and Foster, 2012). They recently explored the model to include adhesion as a key factor. They found that if the host secretes large amount of a matrix, such as mucus, positive selection via adhesion could be transformed into negative selection. The mucus glycans and IgA were experimentally tested in this model (McLoughlin et al., 2016).

An active selection mechanism observed by us, however, does not rely on the AMPs or adaptive immune response. Others and we found abundant of microRNAs in the animal gut lumen and feces (Ahmed et al., 2009; Link et al., 2012; Liu et al., 2016). We found that the microRNAs in the gut lumen were actively generated by the host intestinal epithelia (Liu et al., 2016). These microRNAs were able to enter bacteria and regulate bacterial gene expression. Interestingly, such regulation can be either up-regulating or down-regulating. We observed that this inter-kingdom regulation was important for the establishing of a core microbiota. Mice deficient of fecal microRNA were not able to establish a core gut microbiota, while receiving fecal microRNA from wild type mice was able to re-establish a microbiota resembling that of wild type (Liu et al., 2016). The detailed regulating mechanisms are yet to study. One possible transfer mechanism is through extracellular vesicles (EV), as we observed abundant of EV in the feces containing microRNA (Liu et al., 2016). As a membrane surrounded cargo with informative components (lipids, proteins, and RNAs including microRNA), EV is an emerging mechanism bridging interkingdom crosstalk (Celluzzi and Masotti, 2016).

## Summary and future direction

**Figure 1:**
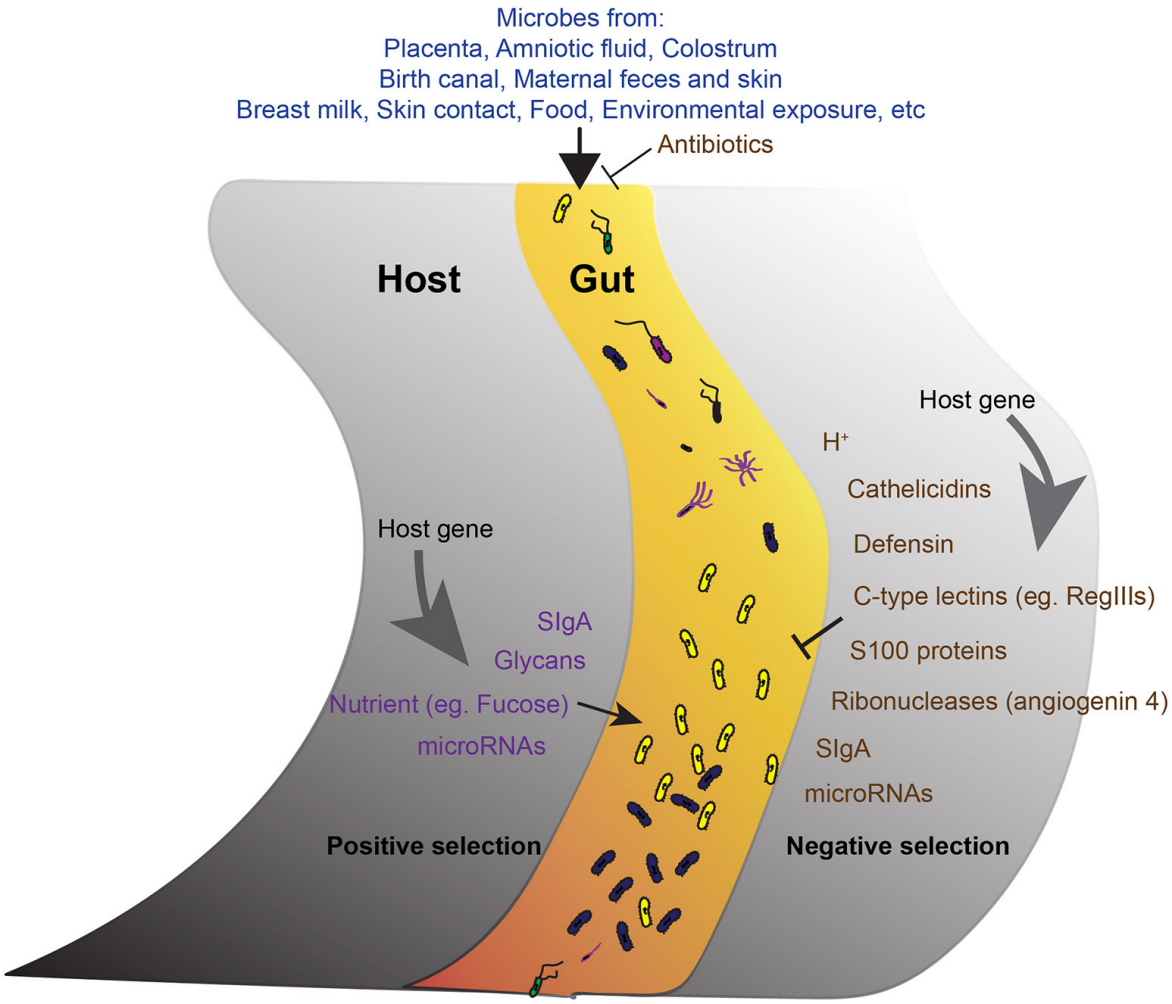
**The development of gut microbiota**. A schematic of the proposed model showing the main factors involve in the development of the core gut microbiota. The acquirement of bacteria from different sources is the initial step. The microbes get into the gut then undergo both negative **(right)** and positive **(left)** selections from the host. Antimicrobial activities reduce unfavorable microbes. Positive selection promotes the abundance of favorable commensals. Host genetic background and host gene expression are important factors endorse the selection.

Gut microbiota has been proved to be important in health and its dysfunction has been linked to many diseases. Different diseases have been associated with changes in different microbial species. Current available antibiotics generally non-specifically eliminate large spectrum of bacteria, which is a disaster for gut commensals and public health as increasing antibiotic-resistant pathogens emerge. Fecal microbiota transplantation (FMT) is undergoing trial for the treatment of *Clostridium difficile* infection not responding to standard therapies. However, besides lack of specificity, the use of FMT in other diseases faces biosafety concerns (Ianiro et al., 2014). These call for more detailed studies on species- and gene- specific manipulation. As one side of host-microbe crosstalk, the mechanisms of how host shapes its gut microbiota (Figure 1) provide strategies to improve gut health. Particularly, the active positive selection mechanisms provide a promising species-specific strategy. However, the detailed molecules involved in these mechanisms; how each bacterial species can be manipulated; and how the community as a whole can be manipulated for the wellness of health require future study.

## Author contributions

SL conceived the concept and wrote the manuscript.

### Conflict of interest statement

The author declares that the research was conducted in the absence of any commercial or financial relationships that could be construed as a potential conflict of interest. The reviewer AC and handling Editor declared their shared affiliation, and the handling Editor states that the process nevertheless met the standards of a fair and objective review.
